# Combined Assessment of Gastrointestinal Hormones and Metabolomic Profiling Following Mixed-Meal Tolerance Tests in Patients at Increased Risk of Refeeding Syndrome: Study Protocol

**DOI:** 10.3390/jpm16090452

**Published:** 2026-08-28

**Authors:** Gonçalo Nunes, Marta Guimarães, Sofia S. Pereira, Ivo Mendes, Francisco Vara-Luiz, Cátia Oliveira, Marta Gonçalves, Patrícia Mendes, Rute Santos, Tânia Meira, Jorge Fonseca

**Affiliations:** 1 Gastroenterology Department, GENE—Artificial Feeding Team, Unidade Local de Saúde Almada-Seixal, Hospital Garcia de Orta, 2805-267 Almada, Portugal; 2 ICBAS-UP—School of Medicine and Biomedical Sciences, University of Porto, 4050-313 Porto, Portugal; 3 Aging Lab, Egas Moniz Center for Interdisciplinary Research (CiiEM), Egas Moniz School of Health & Science, 2829-511 Almada, Portugal; 4 Department of Endocrine and Metabolic Research in Biomedicine, ICBAS—School of Medicine and Biomedical Sciences, University of Porto, 4050-313 Porto, Portugal; 5 ITR—Laboratory of Integrative and Translational Research in Population Health, Rua das Taipas 135, 4050-600 Porto, Portugal; 6 Clinical Pathology Department, Clinical Chemistry Laboratory, Unidade Local de Saúde Almada-Seixal, Hospital Garcia de Orta, 2805-267 Almada, Portugal

**Keywords:** refeeding syndrome, gastrointestinal hormones, metabolomics, mixed-meal tolerance tests, PEG

## Abstract

**Introduction:** Refeeding syndrome (RS) is a life-threatening metabolic complication of nutritional support. Prolonged fasting, frequently observed in malnourished patients referred for percutaneous endoscopic gastrostomy (PEG), may induce histological and ultrastructural changes in the intestinal mucosa, potentially influencing metabolic adaptation during nutritional reintroduction. Mixed-Meal Tolerance Tests (MMTT) combined with targeted metabolic profiling allow dynamic assessment of serum glucose, gastrointestinal hormones and metabolites, which may help to elucidate the metabolic adaptations associated with fasting and refeeding. **Objective:** The present study aims to perform MMTT in PEG patients to characterize enteroendocrine hormone responses and metabolomic profiles following a prolonged period of reduced nutritional intake and subsequent enteral refeeding. **Methods:** This prospective, single-center study includes adults referred for PEG after at least one month of oral intake below 50% of energy needs. The MMTT will be performed at PEG placement and after 3–6 months of enteral nutrition. Serial blood samples will be collected from baseline up to 120 minutes post-meal to measure serum glucose, insulin, C-peptide, electrolytes and gastrointestinal hormones (GLP-1, GIP, ghrelin and PYY), and for targeted metabolomic profiling by spectroscopy. Clinical and nutritional data will be prospectively recorded. Exploratory analyses will assess metabolic and hormonal changes over time and their potential associations with relevant clinical characteristics. The study was approved by the institutional ethics committee, and patient informed consent will be obtained. **Conclusion:** This study integrates MMTT and targeted metabolomic profiling to characterize hormonal and metabolic responses during fasting and refeeding in PEG patients considered at increased risk of RS due to prolonged markedly reduced oral intake. The findings may improve understanding of metabolic adaptations associated with nutritional reintroduction and may identify candidate hormonal and metabolic signatures to support future studies on RS pathophysiology and risk assessment.

## 1. Introduction

Refeeding syndrome (RS) is a potentially life-threatening metabolic complication that occurs when nutrition is reintroduced after prolonged fasting or malnutrition, leading to electrolyte imbalances—especially low serum phosphate, magnesium and potassium—and fluid shifts that may cause organ dysfunction. Although RS has been traditionally reported in hospitalized high-risk and critically ill patients, its prevalence is probably underestimated, especially in ambulatory individuals needing artificial feeding [1,2]. More recently, standardized criteria for the diagnosis and staging of RS were proposed by the American Society for Parenteral and Enteral Nutrition (ASPEN), building on previously established criteria for identifying patients at risk of RS, including those described by the National Institute for Health and Care Excellence (NICE) [3,4].

Some studies suggest that refeeding hypophosphatemia is more common in enteral than parenteral feeding in adult hospitalized patients [5]. However, in patients undergoing percutaneous endoscopic gastrostomy (PEG) for long-term enteral nutrition due to chronic disease with prolonged dysphagia, a particular high-risk subgroup, clinical and laboratory signs of RS are seldom identified [6]. In fact, chronic starvation induces histological, ultrastructural and hormonal changes in the duodenal mucosa of patients at increased risk of RS as it was already demonstrated in two recent prospective controlled studies [7,8]. These changes include shortening of the villi with or without mucosal atrophy, ultrastructural evidence of autophagy and increased tissue expression of glucagon-like peptide 1 (GLP-1) and gastric inhibitory polypeptide (GIP) [7,8]. GLP-1 and GIP are the two primary incretin hormones secreted from the intestine after food ingestion to stimulate insulin secretion from pancreatic beta cells [9,10]. This incretin overexpression may constitute an adaptative response to maintain insulinogenic activity for essential anabolic processes during long-term low food supply. Actually, although the impaired absorption expected from the histologic and ultrastructural changes in the duodenum may probably avoid RS, the enteroendocrine adaptative response of the duodenal mucosa may paradoxically increase its risk after enteral PEG refeeding [8].

Gastrointestinal hormones are bioactive peptides released by the gut in response to nutrient ingestion, playing a key role in regulating digestion, absorption, appetite regulation, and systemic metabolic homeostasis [11]. Beyond incretins, ghrelin and peptide YY (PYY) are other gastrointestinal hormones with complementary roles: ghrelin, produced mainly in the stomach, stimulates appetite, whereas PYY, secreted primarily in the distal intestine, promotes satiety and slows gastric emptying, together regulating energy balance and postprandial metabolism [11,12].

Mixed Meal Tolerance Tests (MMTT) are standardized clinical procedures in which a subject ingests a mixed-nutrient meal containing carbohydrates, proteins and fats, followed by serial measurements of serum glucose, insulin and other metabolic hormones over time. These tests are used to assess postprandial metabolic responses, including beta-cell function, insulin sensitivity and gastrointestinal hormone secretion [13,14,15].

Metabolomic analysis is the systematic study of small-molecule metabolites within biological samples, providing a functional snapshot of cellular metabolism and its response to physiological and pathological changes. Targeted metabolic profiling refers to the focused analysis and quantification of a predefined set of metabolites to characterize specific metabolic alterations and biological pathways.

Combining MMTT with metabolomic analysis provides a dynamic framework to investigate the metabolic shifts occurring during nutritional reintroduction. By capturing real-time changes in glucose, insulin, electrolytes, gastrointestinal hormones, and selected metabolites, this approach may provide insights into biochemical pathways potentially involved in metabolic disturbances occurring during nutritional reintroduction, including carbohydrate oxidation, phosphate utilization, and energy metabolism [16,17]. Such integrated functional analysis complements the morphological and immunohistochemical study of the duodenal mucosa and may help to elucidate mechanisms underlying electrolyte imbalances and metabolic stress, providing a basis for future studies of RS-related metabolic disturbances.

### 1.1. Research Questions

(A) What are the kinetics of serum concentration of glucose, insulin, electrolytes and gastrointestinal hormones (GLP-1, GIP, ghrelin and PYY) during fasting and refeeding?
(B) Which serum metabolites may be associated with metabolic adaptations during nutritional reintroduction in a patient population considered at increased risk of RS undergoing enteral nutrition?

### 1.2. Objectives

(A) To define the kinetics of serum glucose, electrolytes and gastrointestinal hormones during fasting and refeeding.
(B) To perform MMTT and metabolomic analysis in patients referred for endoscopic gastrostomy after a significant period of reduced oral intake.
(C) To repeat the same evaluation after 3–6 months of long-term enteral nutrition with adequate energy supply.
(D) To analyze the evolution of serum metabolites during fasting and refeeding and explore potential metabolic signatures that may be relevant to future studies of RS risk.

## 2. Methods

### 2.1. Design

This was a single-center, observational and prospective study conducted in a tertiary hospital.

### 2.2. Population and Recruitment

A convenience sampling approach will be used to recruit ambulatory patients referred for PEG for long-term enteral nutrition. Adult patients (aged ≥ 18 years) with oral intake below 50% of daily energy requirements for a minimum period of 1 month and no previous use of artificial nutrition (oral supplements, tube feeding, or parenteral nutrition) will be eligible to participate in the study. Patients aged below 18 years, with oral intake above 50% of daily energy requirements during the previous month, and/or with oncologic disease, diabetes mellitus, or an expected survival of less than 3 months will be excluded. The inability to provide reliable anamnestic data or to undergo appropriate clinical and nutritional follow-up will also constitute exclusion criteria. The expected indications for PEG in the study population will include chronic dysphagia and inability to maintain adequate oral intake due to neurological disorders or other chronic non-oncological conditions requiring long-term enteral nutrition. Table 1 summarizes the inclusion and exclusion criteria for the study.

### 2.3. Data Collection

For each patient included in the study, several clinical features will be recorded:

1. Gender and age.
2. Height, weight and body mass index (BMI). BMI will be calculated as weight/height^2^ (kg/m^2^) or, if patients could not stand up for height evaluation, BMI will be estimated using the mid-upper arm circumference (MUAC) and regression equations described by Powel-Tuck and Hennessy [18], as this method has been previously used and proved to provide reliable BMI estimation in PEG patients [19].
3. Anthropometry, including MUAC, calf circumference (CC), and triceps skinfold thickness (TSF). Mid-arm muscle area (MAMA) will be calculated from MUAC and TSF.
4. Underlying disease, clinical indication for PEG, and relevant comorbidities.
5. A dietary history referring to the month prior to PEG. Energy-protein requirements will be calculated according to the patient’s clinical data.

Subsequently, the following standardized methodology will be applied:

1. Placement of a peripheral venous catheter with extension line.
2. PEG will be performed by two gastroenterologists using the “pull” method described by Gauderer–Ponsky [20] with patients under deep sedation.
3. The baseline MMTT will be performed on the day of PEG placement. A single fasting blood sample will be collected immediately before sedation and PEG placement, representing the baseline fasting condition. PEG placement will then be performed under deep sedation. After completion of the procedure and once the patient is clinically stable, the standardized test meal will be administered as a bolus through the newly placed PEG tube, representing the first controlled nutritional exposure following the period of reduced oral intake.
4. Blood sampling prior to the procedure for one EDTA K2-Gel tube (7.5 mL) and one serum tube (7.5 mL), corresponding to approximately 15 mL per sampling time point and 105 mL per MMTT session. The serum tube will be used for the required serum assays performed at the hospital clinical pathology department, while the EDTA K2-Gel tube will be used to obtain plasma, which will be stored for subsequent metabolomic analysis at the university research laboratory.
5. The extension must be flushed with heparinized saline between samplings. After completion of the PEG procedure and clinical stabilization, the standardized test meal will be administered as a bolus through the PEG tube over less than 15 min. The same standardized test meal (Fortimel^®^, Nutricia, Portugal; 125 mL, 300 kcal, 18 g protein, 25 g carbohydrates, 9.6 g fat, and 570 mOsm/L) will be administered at both study visits to ensure a reproducible nutrient stimulus and allow within-subject comparison. A weight or energy-adjusted meal was not used to avoid differences in nutrient load between assessments. The relative caloric load may differ between visits according to changes in body weight and nutritional requirements. Individual nutritional status and prescribed and delivered energy intake will therefore be recorded and considered when interpreting the results. Blood samples will then be repeated at 15, 30, 45, 60, 90 and 120 min after the meal, with strict adherence to the scheduled times. Patients should remain supine with the head of the bed elevated at 45 degrees. All samples must be delivered to the laboratory within 30–60 min after collection for further processing. The sedative agents and other medications administered during the PEG procedure, including their doses and administration times, will be prospectively recorded for each participant. The duration of the PEG procedure and the interval between completion of sedation and administration of the test meal will also be recorded and considered when interpreting the postprandial hormonal and metabolic responses.
6. Capillary blood glucose and vital parameters (heart rate and blood pressure) will also be assessed at the same time points. Tolerance to the test meal will be clinically monitored, and symptoms potentially related to meal administration, including nausea, vomiting, diarrhea, dumping-like symptoms or suspected aspiration, will be recorded. If clinically significant intolerance occurs, the MMTT will be interrupted or discontinued according to clinical judgement.
7. EDTA K2-Gel tubes must be kept at 4 °C until centrifugation. Samples should be centrifuged at 2500 g for 12 min at 4 °C. The plasma from each tube should then be pipetted into microtubes of approximately 1 mL (totaling 3–4 mL per time period) and stored at −80 °C until analysis. Each tube must be properly labeled with the coded patient identifier and the corresponding sampling time point for further data registration.
8. Serum assays performed in the hospital laboratory will include measurements of glucose, serum electrolytes (phosphorus, magnesium and potassium), insulin, c-peptide, GLP-1, GIP, ghrelin and PYY at each sampling point. Hormonal assays will be performed using validated methods, with the specific analyte forms and assay platforms defined according to the validated methods available at the time of analysis. The tubes designated for metabolomic analysis after initial centrifugation should be frozen as explained above until transportation to ICBAS-UP for subsequent analysis.

Blood sampling will be performed only when considered clinically appropriate, and the MMTT may be postponed, interrupted or omitted if the patient’s clinical condition makes repeated blood sampling inappropriate.

MMTT will be performed in all patients at the time of PEG placement (baseline) and repeated after 3–6 months of ambulatory enteral nutrition, allowing sufficient time for nutritional rehabilitation while maintaining feasibility in this clinically fragile population. The exact timing of the second evaluation will depend on individual clinical evolution and routine outpatient follow-up. Importantly, the objective of this second assessment is not to compare different follow-up durations, but rather to evaluate changes in hormonal and metabolic responses after a period of sustained nutritional support. During the 3–6-month follow-up period, each patient will undergo an individualized nutritional plan calculated by the dietitian and administered through the feeding tube, aiming to achieve the estimated energy and macronutrient requirements according to the underlying clinical condition. The prescribed nutritional plan, delivered energy and protein intake, enteral formula characteristics, use of oral nutritional supplements, feeding interruptions, adherence, and changes in anthropometric parameters will be recorded.

In addition, longitudinal clinical variables that may influence hormonal or metabolic responses will be prospectively recorded, including changes in the underlying disease, major intercurrent events or hospitalizations, PEG-related complications, and medication initiation, discontinuation, or dose changes. Particular attention will be given to medications potentially affecting glucose metabolism, insulin secretion, gastrointestinal hormone secretion, or nutritional status, including corticosteroids, GLP-1 receptor agonists, somatostatin analogues, prokinetic agents, proton pump inhibitors, and antipsychotics. The clinical, medication, and nutritional status of each patient will be documented at the second MMTT.

Given the exploratory and hypothesis-generating nature of the study, the planned sample size of 15 participants refers to patients completing both MMTT assessments (baseline and 3–6 months). This sample size was defined primarily according to feasibility considerations, previous experience from related studies conducted by our group, and the specific challenges associated with recruiting and longitudinally following patients at increased risk of RS. No formal sample size calculation was performed because the study is intended to generate preliminary mechanistic data and characterize longitudinal hormonal and metabolic responses, thereby supporting the design of future adequately powered studies. Patients who do not complete the follow-up MMTT will not be considered part of the final longitudinal study population.

**Figure 1:**
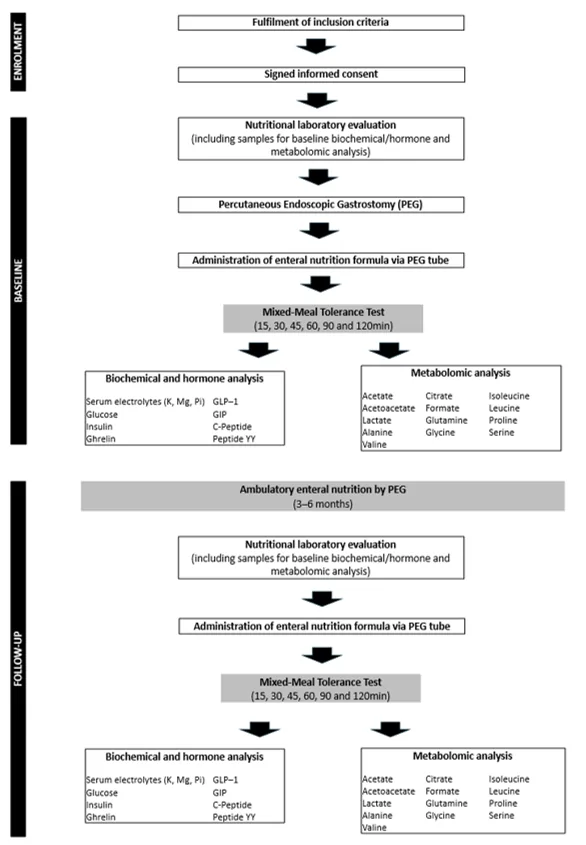
Study flow diagram.

The patient flowchart is described in Figure 1.

### 2.4. Statistical Analysis

The statistical analysis will be performed using the Statistical Package for Social Sciences (IBM SPSS^®^ Statistics, version 29.0). Continuous variables will be expressed as the mean and standard deviation or medians and interquartile ranges, according to data distribution. Categorical variables will be reported as total and relative frequencies. Normality will be assessed using the Kolmogorov–Smirnov test.

Given the biomarker-oriented design of the study, no single primary endpoint will be defined. Hormonal, biochemical, and metabolomic variables will be evaluated as complementary markers of fasting and postprandial metabolic adaptation. Inferential analyses will be performed to evaluate changes in serum concentrations of electrolytes, glucose, and gastrointestinal hormones during the Mixed Meal Tests, as well as differences between baseline and the evaluation performed after 3–6 months of enteral nutrition. Paired statistical tests appropriate to data distribution will be applied for comparisons between time points and between study visits. The overall temporal profiles across the predefined postprandial time points will be considered in the interpretation of the findings.

Exploratory analyses will be performed to investigate associations between hormonal/metabolic profiles and relevant clinical characteristics, including nutritional status and patient survival. Potential confounding variables, including underlying disease, PEG indication, medication use, and relevant clinical/laboratory characteristics, will be considered during data interpretation.

Given the limited sample size and the large number of biomarkers and repeated measurements, statistical findings will be interpreted cautiously. Effect sizes and confidence intervals will be considered alongside *p*-values, which, when reported, will be regarded as descriptive rather than confirmatory. No formal multiplicity-adjusted analysis will be performed, as the study is intended to characterize patterns of hormonal and metabolic responses and to generate hypotheses for subsequent studies. Multivariate analyses will likewise be considered exploratory, with principal component analysis (PCA) used mainly for visualization of metabolic patterns and partial least squares discriminant analysis (PLS-DA) interpreted cautiously due to the potential risk of overfitting. Findings from multivariate analyses will require validation in larger independent cohorts.

Potential attrition between the baseline and follow-up assessments is anticipated because of disease progression, mortality, PEG-related complications, or loss to follow-up. All available baseline data will be included in descriptive analyses. Longitudinal paired analyses will be based on participants with available measurements at both study visits. The number and reasons for missing follow-up assessments will be explicitly reported. No imputation of missing biological measurements will be performed. The potential impact of attrition and incomplete paired observations will be considered when interpreting the results.

### 2.5. Metabolomic Analysis

A targeted metabolic profiling approach will be performed, focusing on a predefined panel of metabolites involved in energy metabolism, amino acid metabolism, and substrate utilization pathways relevant to nutritional deprivation and refeeding.

Plasma samples will be mixed and incubated with cold methanol (1:2 *v*/*v*) for protein precipitation. After that, samples will be centrifuged and the supernatant collected and dried in a vacuum concentrator. Samples will then be resuspended in a fumarate solution in D_2_O (2 mM, pH 7). Sodium fumarate (2 mM; 6.50 ppm) will be used as an internal reference. Spectra will be acquired using NOR5X3INSB optimizer inserts (Norell, Landisville, NJ, USA) on a Bruker Ascend 600 MHz Avance III HD spectrometer equipped with a 5 mm CPP BBO 600S3 BB-H F-D-05 Z probe. The Bruker TopSpin® 5.0.0 software (Bruker Corporation, Billerica, MA, USA) will be used for processing the spectra and metabolite quantification (Acetate, Acetoacetate, Alanine, Citrate, Creatine, Formate, Glutamine, Glycine, Isoleucine, Lactate, Leucine, Proline, Serine, Valine). Peaks will be assigned through comparison with reference spectra using Chenomx 12.0 (Chenomx Inc., Edmonton, Canada) and the Human Metabolome Database (HMDB) V 5.0. Initial data structure will be evaluated by principal component analysis (PCA), followed by partial least squares discriminant analysis (PLS-DA) to identify metabolite contributions to class separation. Loadings of pair-wise comparison PLS-DA models will be calculated by multiplying the variable weights (w) with the respective standard deviations and will be color-coded according to the size of variable importance on projection (VIP) values. VIP > 1 will be considered relevant to group separation.

### 2.6. Ethics and Confidentiality

This research will be conducted in accordance with the Declaration of Helsinki and was reviewed and approved by the Hospital Garcia de Orta Ethics Committee (approval code 02/2024). The study protocol was developed and reported in accordance with the SPIRIT 2013 recommendations, and the completed SPIRIT checklist is provided as supplementary material. All participants or their caregivers must agree to all study procedures and sign the informed consent form. Participation will be entirely voluntary, and patients may withdraw from the study at any time. All data collected from participants will be strictly anonymous and confidential.

## 3. Discussion

The current study aims to perform MMTT and metabolomic analysis to patients referred for PEG with chronic reduced ingestion or established malnutrition, a population considered to be at increased risk of RS, in order to define the evolution of serum glucose, insulin, electrolytes, gastrointestinal hormones (GLP-1, GIP, ghrelin and PYY) and several cellular metabolites during fasting and immediate refeeding. This analysis will contribute to unveiling some of the underlying pathogenic mechanisms involving RS, especially with regard to enteroendocrine adaptative changes, to identify candidate hormonal and metabolic signatures that may contribute to future biomarker discovery, and to optimize patient risk stratification and overall clinical management. The repetition of both analyses in each patient after 3–6 months of adjusted enteral nutrition will enable comparison between the period of prolonged nutritional deprivation and the period of adequate intake following the nutritional intervention, thereby allowing assessment of changes in hormonal and metabolic responses following nutritional rehabilitation. Indeed, the current strategies used in clinical practice to stratify the risk of RS are becoming clearly insufficient, as several studies have reported their limited accuracy in predicting this condition [5]. Most of these criteria rely on body mass index, unintentional weight loss, duration of nutritional deprivation, low serum electrolyte concentrations (potassium, phosphorus, and magnesium), and a history of alcohol use or specific drug consumption. More recent evidence has incorporated body composition-based criteria derived from bioimpedance analysis and considers underlying diseases or comorbidities in patient risk stratification [3,4]. The identification of hormonal or metabolite patterns may contribute to a better understanding of metabolic adaptations associated with refeeding risk and may support the development of future studies focused on improved patient stratification.

Previous studies conducted by our group demonstrated that prolonged fasting induces alterations in the duodenal mucosa, including changes in the tissue expression of gastrointestinal incretins, with potential implications for immediate nutrient absorption following nutritional reintroduction and for the development of RS [7,8]. The current protocol will include a functional analysis aimed at determining whether the previously observed immunohistochemical patterns correlate with serum concentrations after a controlled “refeeding simulation”. We hypothesize that serum GLP-1 and GIP concentrations may increase after refeeding, potentially reflecting the previously reported accumulation of both incretins within intestinal cells during prolonged fasting [8]. A similar response may be observed for PYY, whereas an inverse response may occur for ghrelin, whose serum levels typically decrease following meal ingestion [11].

In patients at increased risk for RS, targeted metabolite profiling may identify metabolic patterns consistent with prolonged catabolic adaptation and impaired substrate flexibility, including increased levels of ketone bodies such as acetoacetate, alterations in amino acid pools involved in gluconeogenesis and protein turnover like alanine, glutamine, glycine, serine and proline, disruptions in branched-chain amino acids such as leucine, isoleucine and valine, and shifts in intermediates of energy metabolism such as citrate, lactate, formate and creatine. Together, these disturbances reflect impaired oxidative capacity and sustained reliance on fat and protein breakdown prior to refeeding, thereby providing a biochemical signature that may help to identify patients most vulnerable to metabolic collapse during early nutritional rehabilitation [21,22].

After 3–6 months of enteral nutrition via PEG, we hypothesize that the postprandial hormonal and metabolic response may differ from that observed at baseline, potentially reflecting partial normalization of the adaptations associated with prolonged nutritional deprivation. This hypothesis is supported by our previous findings showing normalization of histological and ultrastructural alterations in the duodenal mucosa after a comparable period of nutritional support [7]. However, the magnitude and direction of these changes may also be influenced by differences in nutritional status, disease evolution, medication exposure, and the relative energy load of the fixed test meal at the second assessment. Therefore, an attenuated response at follow-up should be considered a hypothesis rather than a predefined outcome.

This study protocol has some limitations that should be acknowledged. The main weakness is its uncontrolled design. Indeed, obtaining an appropriate control group to perform the MMTT in metabolically healthy individuals is challenging, both for ethical reasons and because of the inherent variability in normal postprandial hormonal and metabolic responses. To mitigate this issue, each patient will serve as his or her own control, with baseline results compared to those obtained after a period of presumed adequate nutrition during the second evaluation. Although this within-subject design reduces interindividual variability, several factors including age, underlying disease, inflammatory status, medication exposure, and PEG indication may influence hormonal and metabolomic responses. These variables will therefore be considered during data interpretation. The longitudinal nature of the study also introduces potential sources of variability between the two study visits. In particular, interindividual differences in patients’ diets may influence the observed hormonal and metabolic responses. Standardizing dietary intake over a 3–6-month period would be virtually impossible unless nutrition were exclusively formula-based, which would be socially and economically unsustainable and would not reflect the real-life clinical management of PEG-fed outpatients. Furthermore, changes in the underlying disease, medication exposure, inflammatory status, nutritional intake, and PEG-related factors during the 3–6-month follow-up may influence gastrointestinal hormone and metabolite concentrations independently of nutritional rehabilitation. Although these variables will be systematically recorded and considered in the interpretation of the results, their effects cannot be completely separated from those of nutritional rehabilitation in this uncontrolled observational design. The MMTT procedure itself may also introduce some sources of variability. The use of the same fixed test meal at both study visits represents a potential limitation, as its relative caloric load may differ according to changes in body weight, nutritional status, and energy requirements after nutritional rehabilitation. In addition, the fasting baseline sample is collected before sedation and PEG placement, whereas postprandial samples are collected after the procedure; therefore, the potential effects of sedation, gastric insufflation and procedural stress on subsequent hormonal and metabolic responses cannot be completely excluded. Sedative agents and their timing will be recorded and considered when interpreting the results. Gastric emptying was not directly assessed and may also influence postprandial glucose and gastrointestinal hormone kinetics, particularly given the potential differences between the baseline and rehabilitated nutritional states. A further limitation is that clinically defined RS episodes will not be prospectively ascertained as a study outcome. Therefore, the present study cannot determine whether specific hormonal or metabolomic responses are associated with the subsequent development of clinically diagnosed RS. Instead, the study is intended to generate mechanistic data in a population considered at increased risk based on prolonged markedly reduced nutritional intake. Finally, the small sample size and potential attrition during the 3–6-month follow-up may reduce statistical precision and limit the generalizability of the findings. The study should therefore be regarded as exploratory and hypothesis-generating, with its results intended primarily to inform the design of future larger and adequately powered studies.

In conclusion, the present study integrates the assessment of gastrointestinal hormones and metabolomic profiling following MMTT in patients at increased risk for RS, due to prolonged markedly reduced oral intake. To the best of our knowledge, this is the first study to apply this methodology in this specific patient population, aiming to elucidate the enteroendocrine and metabolic responses to fasting and refeeding. These findings are expected to complement morphological and immunohistochemical analyses and may contribute to advancing and redefining current approaches to RS risk evaluation.

## Figures and Tables

**Table 1:** Summary of patient inclusion and exclusion criteria.

Inclusion Criteria
Age above 18 years.
Oral ingestion below 50% of energy daily needs for ≥ 1 month.
Absence of artificial nutrition before PEG placement.
**Exclusion Criteria**
Age below 18 years.
Quantified food ingestion above 50% of energy daily needs during the last month.
Oncologic disease.
Diabetes mellitus.
Expected survival below 3 months.
Inability to provide credible anamnestic data.
Impossibility to have an appropriate clinical and nutritional follow-up.

## Data Availability

The original contributions presented in this study are included in the article. Further inquiries can be directed to the corresponding author.

## Supplementary Materials

The following are available online at https://www.mdpi.com/article/10.3390/jpm16090452/s1, SPIRIT 2013 Checklist: Recommended items to address in a clinical trial protocol and related documents.
